# Items

**Published:** 1879-01

**Authors:** 


### Items.

ON THURSDAY, DEC. 19TH, Professor Gunn, after a severe and protracted illness, made his first appearance before the class at Rush Medical College. The Professor was too much prostrated to make any attempt at lecturing, and therefore contented himself with greeting his friends.

In honor of the event, the class had determined upon a welcome with floral decorations. The upper lecture room, where the professor is accustomed to deliver his valuable surgical lectures, was most tastefully decorated with plants, flowers and evergreens. The blackboard, where usually can be found the announcements respecting "material for class," etc., was covered with white cloth, and trimmed with evergreens and holly. In the centre was the word "Congratulamus" in large evergreen letters. Above the blackboard the wall was also tastefully decorated with evergreens and holly. In the centre—in its accustomed place—was the bust of Dr. Gunn's predecessor, Professor Brainard. Immediately at the foot of the middle aisle, upon the rail, was placed an elegant stand of flowers, the base of which was about six feet in circumference—a solid mass of camelias, tuberoses and carnations. Rising from this was a large basket of flowers pyramidal in shape, surmounted by a lilly, with camelias and tuberoses thickly scattered around it. A small wire stem, tastefully decorated, and rising from the main basket, supported the monogram M. G. At the foot of each of the two other aisles was placed a large basket of flowers, somewhat similar to the first, but not so large. Two baskets, of intermediate size, were placed upon the shelves or tables at the sides of the arena. Plants were thickly scattered about, and the place had an appearance so very unusual that probably none of the professor's clinical patients who visit him there on Saturday afternoons, would have recognized it as the arena where epithelioma, necrosed bone, etc., had so often yielded to the touch of the operator.

Mr. Miller, the class president, welcomed the professor in a few well chosen words, expressing the delight of the members of the class in receiving their honored professor, after his narrow escape from the "king of terrors," and presented to Dr. Gunn the main stand of flowers.

The professor's strength would not permit an extended reply, and after expressing his thanks to the class in a very appropriate manner, he withdrew.

Three cheers were then proposed, and the proposition was most heartily responded to.

The class then dispersed, to meet again at 4 o'clock to listen to Prof. Parkes, who has so ably filled the chair of surgery during the absence of its regular occupant. L. C. W.

JUSTICE TO DR. SIMS.—Several medical periodicals in this country have lately published paragraphs containing statements similar to the following, which we find in the *Detroit Lancet*, of Nov., 1878 :

"A correspondent of the CHICAGO MEDICAL JOURNAL states that all the patients Dr. J. Marion Sims operated on while in Vienna, died within ten days of peritonitis."

It was the *Boston Medical and Surgical Journal*, we believe, that first gave prominence to this item, and that subsequently explained the manner in which the error was committed, by which an unnecessary reflection was cast upon Prof. Sims' skill as an operator.

Our Vienna correspondent, writing under date of July 15, 1878 (see Sept. No. of CHICAGO MEDICAL JOURNAL AND EXAMINER, p. 295) uses the following language :

"Among these (operations) he (Dr. Sims) did three for the removal of cancerous portions of the womb by a method called by the Germans the "Trichterförmige Excision," which consists in cutting away with the scissors, a funnel-shaped portion of the cervix uteri, extending, when necessary for the removal of the diseased tissue, into the body of the womb itself. In fact, as done by Dr. Carl Braun, the procedure consists in removing the whole lower portion of the muscular tissue of the uterus, close upon the edge of the peritoneum. Dr. Sims' patients operated upon here, all died of peritonitis within ten days of the operation."

In other words, the three operations, all done for cancerous disease and all involving the tremendous risks of the grave Trichterförmige Excision, terminated fatally.

The great difference between this statement and the isolated sentence which is going the rounds of the medical press, must be apparent to all. Justice to our eminent countryman, now abroad, should lead all our contemporaries who have made the blunder to retract their statements.

Mr. Spencer Wells.—We have recently published a series of papers detailing results in ovariotomy by British and foreign surgeons, such as may well make all Englishmen glad and proud.

If ovariotomy is now, as the results of Spencer Wells, Keith, Nussbaum and Knowsley Thornton show it to be, an operation which is not so hopeless and desperate as to seem unjustifiable even in the face of surely impending death from ovarian disease; if it is now an operation which gives but two deaths in forty-nine cases (and these two cases specially and palpably unfavorable for operation), that grand achievement of science and magnificent boon to humanity is due mainly to the ceaseless exertions of a British surgeon, Mr. Spencer Wells. His name, illustrated by such associations and bound up by undying recollection with the most splendid progress in modern surgery, needs no other decoration. But how strange a commentary on the principles on which the scanty honors of the state are distributed among members of our profession, that it should stand thus undignified in the sense in which alone sovereigns and states can dignify the princes of science or recognize their enduring services to mankind. Had Mr. Wells improved some engine of destruction, he might long since have been gazetted K.C.B. Had he amassed great wealth by selfish means or for selfish ends, he might have been ennobled; in the legal profession he would long since have been on the woolsack, or in the church a spiritual lord. Had his services, of however ordinary a kind, been to the court even in his own noble profession, he could hardly have been long left undistinguished. But, as it is, the name which is throughout the world one of the chief glories of England, as a benefactor to humanity by a specially valuable and touching addition to our means of saving much-loved lives, is also a standing reproach to the indifference with which the highest kind of achievement in life-saving is regarded in governing circles and by the advisers of the crown in the distribution of the honors of which it is the source.—*British Medical Journal.*

IT is generally known to the medical profession and those interested in bibliography that Dr. John S. Billings, Surg., U. S. A., in charge of the National Medical Library, at Washington, is now ready to print his great "National Catalogue of Medical Literature," as soon as Congress grants an appropriation for the purpose. This indexes under subjects, and by authors, books, pamphlets and original papers in nearly all the medical perodicals of the world; including over 400,000 subject entries, and making ten volumes royal 8vo of 1,000 pages each. This will be of the greatest value to physicians the world over, as it enables them to find analogues for peculiar and difficult cases, and thus often to save lives. In continuation of this work, it is now proposed to publish monthly, under the editorship of Dr. Billings and of his assistant, Dr. Robert Fletcher, M. R. C. S., a current medical bibliography under the title of the *Index Medicus.* It will be issued by F. Leypoldt, the bibliographic publisher, 37 Park Row, New York, at $3 per year, and will enter all medical books and index the leading medical journals and transactions in English and other languages. A full list of the latter, numbering over 600, will form a part of the specimen number of the *Index,* soon to be issued.

SUITS for malpractice are so common as to constitute an epidemic in the State of New York.—*Buffalo Med. and Surg. Jour.*

THE science of the physician is above the assertions of the patient.—*Ricord.*

